# Mapping FACT-P to EQ-5D in a large cross-sectional study of metastatic castration-resistant prostate cancer patients

**DOI:** 10.1007/s11136-014-0794-5

**Published:** 2014-10-19

**Authors:** J. Diels, P. Hamberg, D. Ford, P. Wheatley Price, M. Spencer, R. N. Dass

**Affiliations:** 1 Janssen Pharmaceutica NV, Beerse, Belgium; 2 Janssen EMEA, Beerse, Belgium; 3 Department of Medical Oncology, Sint Franciscus Gasthuis & Prostate Cancer Center, Rotterdam, The Netherlands; 4 Oncology, Queen Elizabeth Hospital, Birmingham, UK; 5 Janssen EMEA, High Wycombe, UK

**Keywords:** EQ-5D, FACT-P, Prostate cancer, Quality of life, Mapping

## Abstract

**Purpose:**

To construct a model to predict preference-adjusted EuroQol 5D (EQ-5D) health utilities for patients with metastatic castrate-resistant prostate cancer (mCRPC) using the disease-specific health-related quality of life (HRQoL) measure, functional assessment of cancer therapy-prostate (FACT-P).

**Methods:**

HRQoL data were collected from patients with mCRPC who were enrolled in an observational study conducted in 47 centers across six European Union countries. Utility values were generated using a UK-specific EQ-5D value set. The predictive validity of the five FACT-P subscales, patient demographics, comorbidities and prior chemotherapy was tested using ordinary least squares (OLS), median, Gamma and Tobit multivariate regression models.

**Results:**

FACT-P and EQ-5D questionnaires were completed by 602 (86 %) patients. Mean age [standard deviation (SD)] was 72.1 (7.9) years, mean time from diagnosis (SD) was 5.4 (4.4) years, and mean time since failure of androgen deprivation therapy (SD) was 1.0 (1.6) years. At study inclusion, 39 % of patients were chemotherapy-naïve, 37 % were undergoing chemotherapy, and 24 % were post-chemotherapy. Mean FACT-P and EQ-5D utility values were 104 and 0.66, respectively. OLS regression was the best-performing model, explaining 61.2 % of the observed EQ-5D variation. All FACT-P subscales were significantly predictive; the physical and functional well-being subscales had the highest explanatory value (coefficient 0.023 and 0.001, respectively, *p* < 0.0001). The other variables did not add additional explanatory value.

**Conclusions:**

The algorithm developed enables translation of cancer-specific HRQoL measures to preference-adjusted health status in patients with mCRPC. The function may be useful in calculating EQ-5D scores when EQ-5D data have not been gathered directly.

## Introduction

Prostate cancer is the most common cancer in Europe among men [1]. In 2012, there were 417,000 new cases of prostate cancer in Europe, representing 12.1 % of all new cancers [1]. The economic burden associated with this high incidence is substantial. For example, the combined cost of direct healthcare, informal care and productivity loss associated with prostate cancer was estimated at €7,848 million in the European Union, in 2009 [2].

Despite 80–90 % of metastatic prostate cancer patients responding to androgen deprivation therapy [3], progression to castration-resistant disease occurs in most patients after 2–3 years, with a subsequent survival time of 24–48 months [4]. The health-related quality of life (HRQoL) of patients with prostate cancer declines substantially toward the end of life [5]. Therefore, treatment of metastatic castration-resistant prostate cancer (mCRPC) is mainly palliative, with the aim of prolonging survival, relieving symptoms and improving HRQoL. The European Association of Urology guidelines recommend docetaxel as first-line chemotherapy, together with corticosteroids, for the treatment of symptomatic mCRPC [6]. Bisphosphonates are prescribed for the management of metastatic bone disease (present in >90 % of patients with mCRPC [7]) to prevent skeletal-related events and improve symptom control [6]. Radionuclides, radiotherapy and analgesics may also be considered for the management of bone pain [6].

Cost-efficacy is important in the technical evaluation of new therapies by reimbursement agencies. Generic preference instruments, such as the EuroQol-5D (EQ-5D) [8], can aid decision makers in resource allocation. These instruments generate health state utilities that can be used to compare quality-adjusted life years gained for interventions across different patient groups and diseases. However, measurements of HRQoL in clinical trials often use disease-specific instruments that address outcomes important to a particular patient population, thus limiting their usefulness in cost-utility analyses. One solution is to derive validated algorithms that map scores from disease-specific HRQoL instruments onto generic preference instruments. This approach has been accepted by bodies such as the UK’s National Institute for Health and Care Excellence, who specifically require EQ-5D utility values as part of health technology assessment submissions [9].

In line with such requirements, an increasing number of strategies for mapping disease-specific responses to preference-based instruments have been published. A database held at the Health Economics Research Centre, Oxford University, lists ninety studies of statistical mapping to predict EQ-5D utilities [10]. However, only one of these focused on mCRPC. Furthermore, 17 % of models were based on less than 200 observations and 39 % of studies used ordinary least squares (OLS) regression as the single statistical tool.

The mCRPC study included in the database demonstrated the feasibility of mapping the functional assessment of cancer therapy-prostate (FACT-P) questionnaire, which specifically measures the HRQoL of prostate cancer patients, to EQ-5D scores [11]. However, application of the algorithm to an external data set was found to yield mean EQ-5D values greater than 1 [12], and the algorithm requires a correction applicable to a truncated linear model [13].

Therefore, a requirement remains for the development of a mapping function that adequately predicts EQ-5D utility values based on responses to FACT-P. In this article, we describe the construction of a prediction model using data obtained from a large, cross-sectional, observational study in patients with mCRPC. Furthermore, we assess the performance of four regression models to predict EQ-5D utility values from responses to the FACT-P questionnaire. In addition to OLS, Tobit, median and Gamma regression models were included to account for ceiling effects and to anticipate any violations of normality and homoscedasticity.

## Methods

### Study sample and data collection

Data were derived from a cross-sectional, observational study conducted in six countries: Belgium, France, Germany, Sweden, the Netherlands and the UK. The study enrolled male patients aged ≥18 years presenting with mCRPC at 47 specialist prostate cancer centers during a 10-month recruitment period. Consecutive patients who visited the clinic during regular follow-up visits were invited to participate. Patients were eligible for inclusion in the study if they had a histologically or cytologically confirmed diagnosis of adenocarcinoma of the prostate; prostate cancer progression documented by prostate-specific antigen according to Prostate Cancer Working Group 2 (PCWG2) criteria or radiographic progression, and disease progression despite surgical or medical castration [a testosterone level of <50 ng/dL (<1.735 nM) was required if testosterone levels were routinely measured]. Exclusion criteria included participation in any investigational drug study or any expanded access program during the observation period. Patients’ HRQoL was assessed at the inclusion visit by utilizing the EQ-5D and FACT-P questionnaires.

### Study instruments

FACT-P is a questionnaire that has been validated to estimate HRQoL in men with prostate cancer [14]. The tool comprises the 27-item FACT-General (FACT-G) questionnaire, which measures HRQoL in cancer patients, and a 12-item prostate cancer subscale, designed to measure prostate cancer-specific HRQoL. The FACT-P is scored by adding the subscales of the FACT-G plus the prostate cancer subscale to yield a comprehensive HRQoL score.

The EQ-5D comprises five domains, which measure general health status: mobility, self-care, usual activities, pain/discomfort and anxiety/depression. In this study, the ‘3l,’ rather than the ‘5l’ version of the tool was used, which subdivides each domain into three, rather than five levels. The EQ-5D provides a simple descriptive profile and a single utility index of health status and is widely used in health economic analyses [8].

### Model specifications—statistical analysis

Utility values were derived from EQ-5D profiles based on a UK-specific EQ-5D value set. The mapping exercise was conducted using responses from patients from multiple countries, and UK preference weights were applied.

The predictive validity of the five FACT-P subscales, patient demographics, comorbidities and prior chemotherapy for utility values was tested using four different regression models: (1) OLS regression was used to construct linear prediction models of EQ-5D, describing differences in mean EQ-5D as a function of mean patient characteristics; (2) median regression was used to describe differences in median health status; (3) generalized linear models (GLM) with log link and Gamma family predicting EQ-5D disutility (where disutility = 1—utility), which allows for skewed distribution of utility values and prevents prediction of utilities >1; (4) the Tobit model, also called a censored regression model, designed to estimate linear relationships between variables when there is either left censoring or right censoring in the dependent variable.

### Model validation and predictive ability

A prediction model usually performs better with the data that were used in its development. Therefore, it is critical to evaluate how well the model works with other data sets. Similar to Wu et al. [11], we estimated the cross-validation *R* ^2^ as the primary indicator of prediction model performance. Tenfold cross-validation techniques were employed to derive goodness of fit statistics. To calculate the cross-validation model performance indicators, the study sample was first divided into 10 equally sized groups. Each group was used successively to test each model, and the remaining 90 % of the sample were used to fit the prediction model. The resulting estimated prediction model was then used to estimate the performance of the original 10 % of the sample. Finally, the estimated error terms were pooled to estimate the overall performance of the model. Additionally, the root mean square error and the mean absolute deviation were generated.

Predictive ability was assessed by comparing observed and predicted EQ-5D scores for three patient subgroups that were defined according to the chemotherapy status of patients at study inclusion: chemotherapy-naïve, undergoing chemotherapy and previously treated with chemotherapy.

## Results

### Patient characteristics

The study included 699 patients. Questionnaire response rates were high, and complete FACT-P and EQ-5D questionnaires were available for 602 (86 %) patients. The response rates were not related to any of the baseline characteristics. The baseline characteristics of this population are shown in Table 1. Patient characteristics were generally similar across countries and between chemotherapy status subgroups, except for mean years since prostate cancer diagnosis (7.0, 4.9 and 4.8 years for the post-chemotherapy, chemotherapy-naïve and undergoing chemotherapy groups, respectively) and mean time since mCRPC diagnosis (1.6, 0.7 and 0.9 years, correspondingly), which were higher in patients who had received chemotherapy previously. In the post-chemotherapy group, the median time since the end of chemotherapy was 3.7 months, and in those undergoing chemotherapy, the median time since initiation of treatment was in 4.6 months.

**Table 1:** Patient characteristics at time of inclusion

Number of patients analyzed, *n* (%)	
Total	602 (100)
Germany	272 (45.2)
France	94 (15.6)
Netherlands	89 (14.8)
UK	79 (13.1)
Belgium	45 (7.5)
Sweden	23 (3.8)
Mean age, years (SD)	72.1 (7.9)
Mean time since prostate cancer diagnosis, years (SD)	5.4 (4.4)
Mean time since initiation of androgen deprivation therapy, years (SD)	4.1 (3.5)
Mean time since failure of androgen deprivation therapy, years/diagnosis of mCRPC (SD)	1.0 (1.6)
Treatment status at inclusion, *n* (%)	
Chemotherapy-naïve	236 (39)
Undergoing chemotherapy	223 (37)
Post-chemotherapy	143 (24)
Comorbidity at inclusion, *n* (%)	
Cardiovascular disease	266 (44.2)
Endocrine/metabolic disease	111 (18.4)
Genitourinary disease	88 (14.6)
Renal disease	42 (7)
Gastro-intestinal disease	61 (10.1)
Other	219 (36.7)
No comorbidity reported	114 (18.9)
Gleason score at initial diagnosis	
≤ 5	29 (4.8)
6	51 (8.5)
7	150 (24.9)
8	125 (20.8)
9	129 (21.4)
10	12 (2)
Missing	106 (17.6)

*mCRPC* metastatic castrate-resistant prostate cancer, *SD* standard deviation

Observed mean values for the EQ-5D utility, FACT-P and the five dimensions of the FACT-P are presented in Table 2. For all patients, the mean FACT-P and EQ-5D utility scores were 104 and 0.66, respectively.

**Table 2:** Mean (SE) FACT-P and EQ-5D values

Treatment status	N	PWB	SWB	EWB	FWB	PCS	Total FACT-P	EQ-5D utility
Chemotherapy-naïve	236	21.9 (0.4)	20.7 (0.4)	16.6 (0.3)	17.6 (0.4)	30.1 (0.6)	106.9 (1.6)	0.70 (0.02)
Undergoing chemotherapy	223	20.3 (0.3)	20.9 (0.3)	17.2 (0.3)	15.9 (0.4)	30.1 (0.5)	104.5 (1.4)	0.66 (0.02)
Post-chemotherapy	143	18.8 (0.5)	20.2 (0.5)	16.0 (0.4)	15.4 (0.5)	28.3 (0.7)	98.6 (1.8)	0.60 (0.03)
All patients	602	20.6 (0.2)	20.6 (0.2)	16.7 (0.2)	16.5 (0.3)	29.7 (0.3)	104.0 (0.9)	0.66 (0.01)

*EWB* emotional well-being, *FWB* functional well-being, *PWB* physical well-being, *SE* standard error, *SWB* social well-being, *PCS* prostate cancer subscale

### Model selection

All FACT-P subscales were included individually in the regression model, together with binary comorbidity variables and treatment status (Table 1). Squared terms were entered into each model to allow for nonlinear relationships with utility measures. All variables that were significant at the 0.05 level were retained in the final model.

The OLS model included most covariates of all models and included all FACT-P subscales and age, with quadratic terms for emotional well-being (EWB), functional well-being (FWB) and age. Covariates that did not reach statistical significance were left out of the final model [social well-being (SWB) in the median regression model; SWB, EWB and FWB^2^ in the Gamma model; and FWB^2^ in the Tobit model].

The cross-validation results for all prediction models are presented in Table 3. The OLS, median and Tobit models performed equally well and explained 61.2 % of the variation in the EQ-5D index, while the Gamma-based model explained slightly less variation (59.8 %). OLS regression generated a slightly lower root mean square error, while the mean absolute deviation between observed and predicted values was lowest for the OLS and median regression models.

**Table 3:** Cross-validation results by statistical model

Model	*R* ^2^	Mean absolute deviation	Root mean square error
OLS	0.612	0.148	0.198
Median regression	0.612	0.148	0.201
Gamma	0.598	0.157	0.201
Tobit	0.612	0.149	0.201

*OLS* ordinary least square

Table 4 presents the correlation values between observed and estimated utility values for the different models. The high correlations between estimated values for OLS, median and Tobit regression illustrate that estimates were very similar across the different statistical models.

**Table 4:** Matrix of correlations between observed and estimated utility values, by statistical model

	Observed	OLS	Median	Gamma	Tobit
Observed	1.000	0.789	0.789	0.775	0.790
OLS		1.000	0.993	0.960	0.996
Median regression			1.000	0.965	0.991
Gamma				1.000	0.968
Tobit					1.000

*OLS* ordinary least square

Table 5 shows parameter estimates for the OLS model. All FACT-P scales were found to be significantly predictive with the physical well-being (PWB) (coefficient = 0.022, *p* < 0.0001) and FWB (coefficient = 0.026, *p* < 0.0001) subscales having the highest explanatory value. Nonlinear relationships were observed between age and both the EWB and FWB domains, and this was accounted for by adding a squared term in the regression model. Comorbidities and prior chemotherapy did not add explanatory value.

**Table 5:** Parameter estimates for the OLS model

	Estimate	Standard error	95 % confidence level		*p* value
Intercept	−1.7306	0.458	−2.6283	−0.833	0.0002
Age	0.0384	0.0129	0.0132	0.0636	0.0028
Age^2^	−0.0003	0.0001	−0.0005	−0.0001	0.002
PWB	0.0222	0.0023	0.0176	0.0267	<0.0001
SWB	−0.005	0.0017	−0.0082	−0.0017	0.0026
EWB	0.027	0.0097	0.008	0.046	0.0054
EWB^2^	−0.0007	0.0003	−0.0013	−0.0001	0.0179
FWB	0.0263	0.0064	0.0137	0.0389	<0.0001
FWB^2^	−0.0005	0.0002	−0.0009	−0.0001	0.009
PCS	0.008	0.0016	0.0048	0.0111	<0.0001

*EWB* emotional well-being, *FWB* functional well-being, *OLS* ordinary least squares, *PWB* physical well-being, *SWB* social well-being, *PCS* prostate cancer subscale

**Fig. 1:**
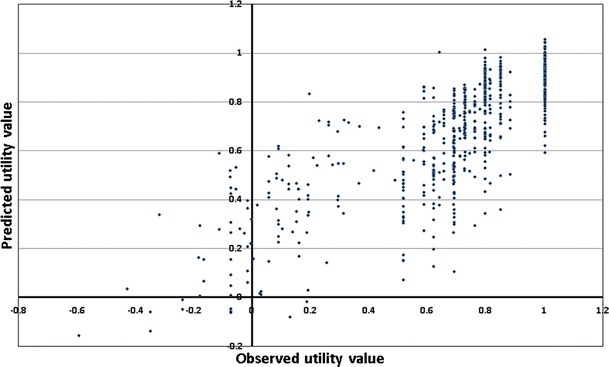
Scatterplot of observed versus OLS-predicted values

Figure 1 shows the scatterplot of observed versus the OLS-predicted values. The predicted utility value exceeded one for a limited number of patients (*n* = 12, 2 %). The highest predicted value was 1.05.

### Predictive ability assessment

**Fig. 2:**
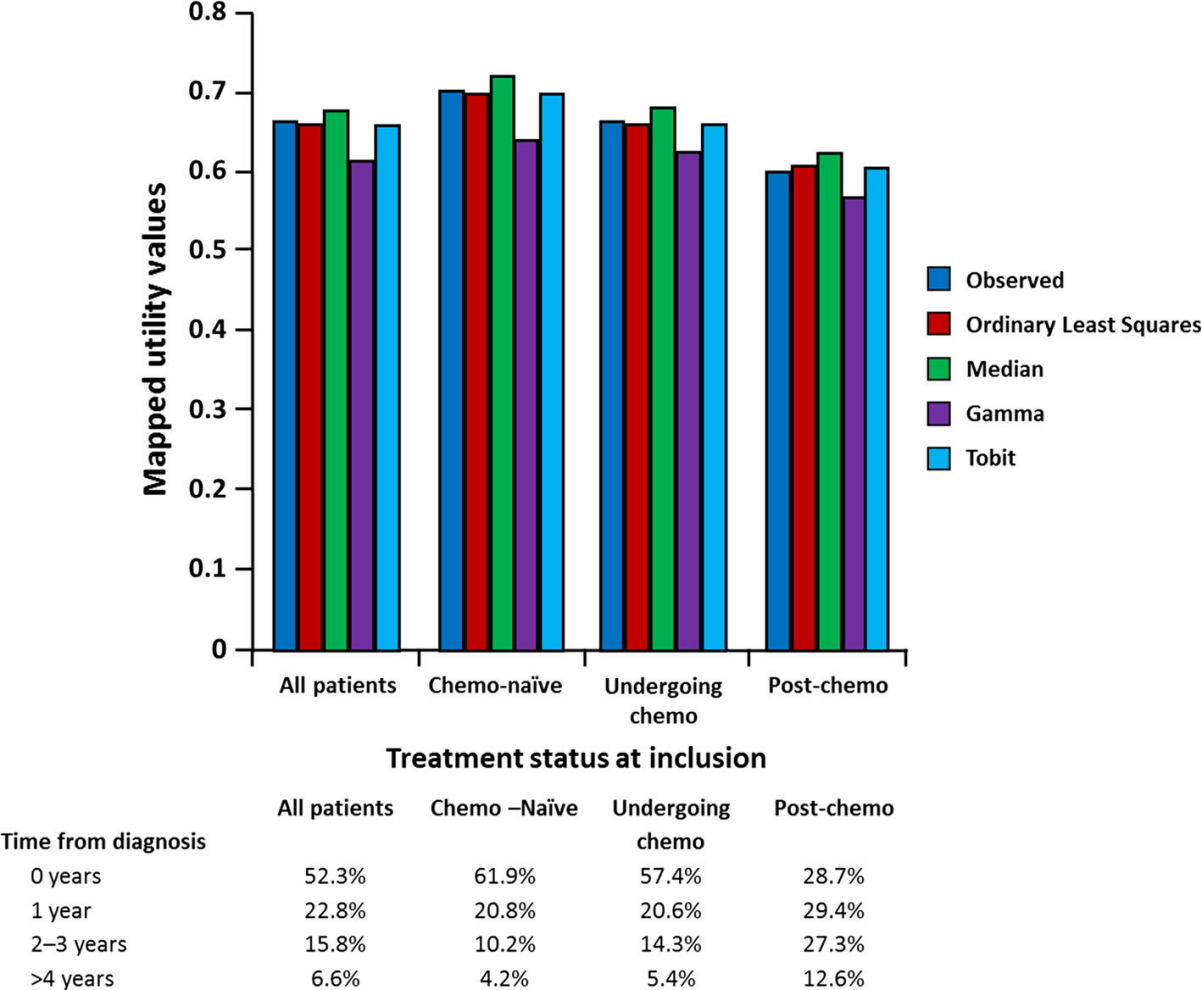
Mean observed and estimated utility values by treatment status at inclusion

**Fig. 3:**
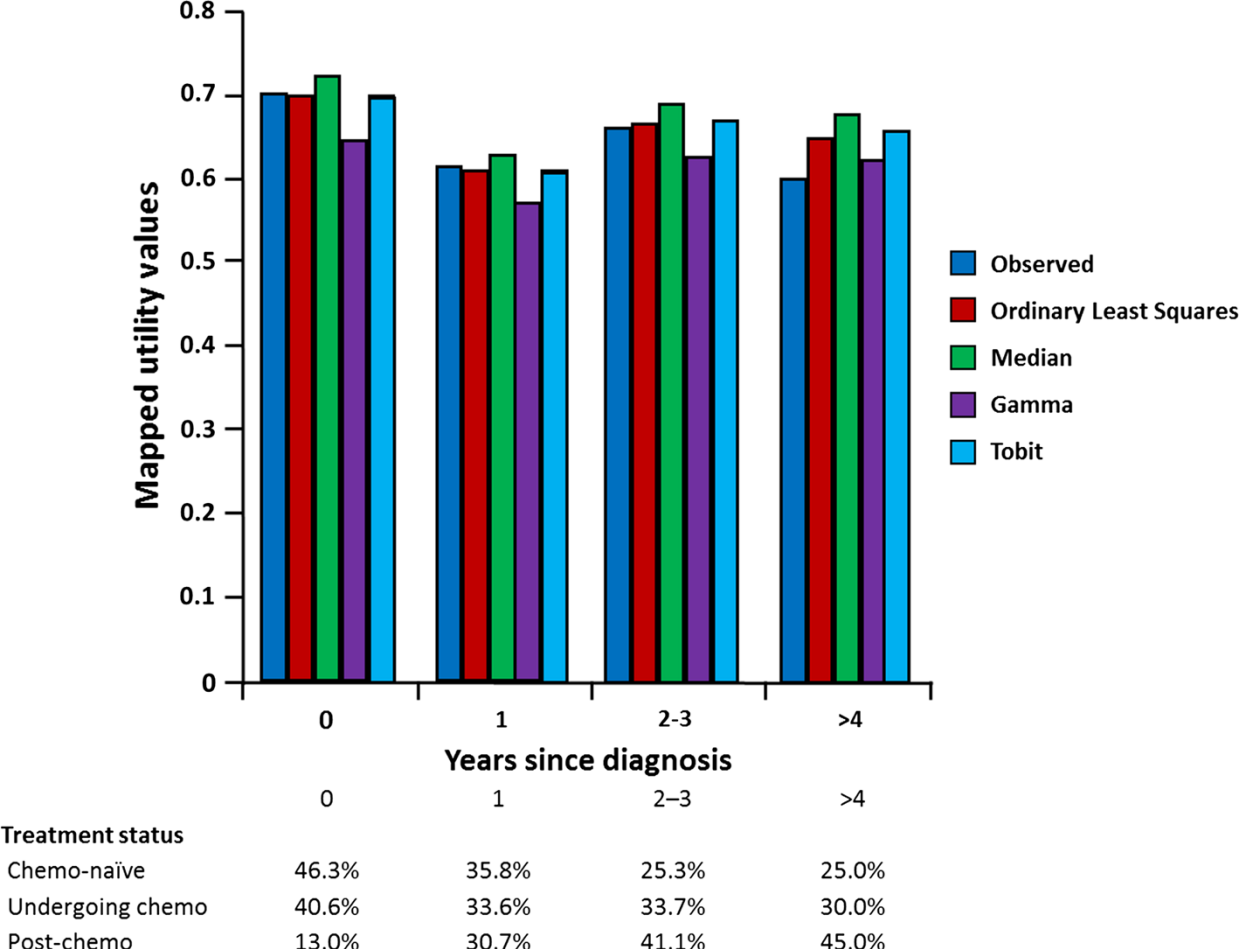
Mean observed and estimated utility values by years since diagnosis

Figure 2 shows the mean observed and estimated utility values as predicted by the four regression models according to treatment status at inclusion. Figure 3 represents a similar graph, stratified by number of years since mCRPC diagnosis. Both graphs suggest that the OLS model provides the best fit for the observed utility values for each of the subgroups. As shown in Table 6, a similar fit between observed and OLS-predicted utility was observed at the country level.

**Table 6:** Mean observed and estimated utility values, by country

Country	Patients (*n*)	Mapped utility value	
		Observed	OLS predicted
Belgium	45	0.62	0.66
France	94	0.62	0.61
Germany	272	0.64	0.63
Netherlands	89	0.75	0.76
Sweden	23	0.78	0.72
UK	79	0.69	0.7

*OLS* ordinary least square

## Discussion

We have developed an algorithm to map FACT-P, a disease-specific instrument, to EQ-5D, a generic preference instrument, based on data collected from mCRPC patients. OLS was the best-performing model. It explained 61.2 % of the variation in EQ-5D values following tenfold cross-validation and provided good concordance between actual and mapped EQ-5D utility scores in predictive assessment.

A previous study demonstrated the feasibility of mapping FACT-P to EQ-5D scores [11]. However, the equation cannot be used without correction [13]. Furthermore, it has been reported that linear regressions may not always accurately predict the EQ-5D distribution for high and low EQ-5D values [15, 16]. Our data tend to confirm this observation; the mapping formula tended to overpredict utility at the lower end of the scale (below 0.4; Fig. 1). Additionally, linear regression may not account for the bounded nature of the EQ-5D, leading to implausible estimates outside of the possible range of values (1 to −0.594). We used a range of regression models to estimate EQ-5D utility values. Tobit regression was included in our analyses to account for the ceiling effect, as it allows for censored dependent variables, and censored the predicted values at 1. However, the Tobit model operates poorly if assumptions of normality and homoscedasticity are violated [17]. Median regression does not rely on these assumptions. However, it has been reported that median regression, while not explicitly dealing with censoring, is equivalent to censored least absolute deviation (used by other mapping studies) when censoring occurs in less than 50 % of the study sample [18]. We also used a generalized linear model (Gamma regression) to account for any skewed distribution of utility values and prevent prediction of utilities >1.

Over the past decade, there has been an increase in the number of studies that have mapped disease-specific responses to preference-based instruments. In addition to the Oxford database [10], a recent literature review identified ten studies that used mapping methods to determine utilities from two cancer-specific instruments (QLQ-C30 and FACT) [19], of which only one study focused on mCRPC patients [11].While statistical models differed across the ten studies, most employed an OLS method and did not conduct an out-of-sample validation. Most studies also used the statistical significance of the coefficients corresponding to different components of the HRQoL scale to determine which variables should be retained in the final model, with a parsimonious approach to final model selection. In doing so, most studies reported that OLS regression performed best, irrespective of its strict assumptions. All of the reviewed studies reported the models’ explanatory power in terms of R^2^, with a range of values between 0.417 and 0.909.

In our study, OLS regression performed equally well to the median and Tobit models in predicting utility scores, with an *R* ^2^ (0.612) in the middle of the range reported by the recent literature review [19]. In view of the relative simplicity of applying OLS regression formulae to other datasets, this was retained as our final model.

The patients included in this study provided FACT-P and EQ-5D data in line with those previously reported. For example, Sullivan et al. measured FACT-P and EQ-5D scores in 280 patients with a mean time from initial diagnosis of prostate cancer to diagnosis of mCRPC of 3.51 years and mean time from diagnosis of mCRPC to study entry of 1.5 years [20]. FACT-P prostate cancer subscale scores ranged from 27.3 to 30.7 and the EQ-5D utility ranged from 0.527 to 0.750 across the seven countries included. These compare to a mean FACT-P prostate cancer subscale of 29.7 and mean EQ-5D utility of 0.66 in our study. In both studies, the FACT-P scores and EQ-5D utility index indicate the significant impact of prostate cancer on patients’ HRQoL.

The mapping exercise in our study was similar to that published previously by Wu et al. [11]. Both studies used data from multinational studies and applied UK preference weights. Mean observed values for EQ-5D and FACT-P were very similar in both studies (EQ-5D:0.66 and 0.64; FACT-P: 104 and 105 for the present study and Wu et al., respectively). In addition to the OLS and median regression employed by Wu et al., we explored two additional statistical models. However, in both studies, OLS was retained as the best-performing model. The estimates of the coefficients of the FACT-P subscales based on the OLS model were in similar directions, with a high weight assigned to the PWB subscale in both studies. However, the larger sample size in this present study (*N* = 602) compared with Wu et al. (*N* = 280) may allow for the generation of more precise parameter estimates.

The limitations of our study include the derivation of utility values using the UK-specific EQ-5D value set. Algorithms developed using country-specific preference weights may account for differences in preferences arising from cultural influences, and value sets should be appropriate to the economic analysis required. The extent to which our algorithm can be generalized is strengthened by the multinational nature of the population that was used. However, further analysis is required to validate the algorithm in other populations.

Although regression-based approaches are commonly used to map HRQol instruments, a recent publication by Fayers et al. (2014) suggests that such approaches may result in biased estimates as a result of regression to the mean [20].

Disease-specific instruments have been developed to address aspects of health-related outcomes that are important to specific patient populations and can overcome the limitation of generic instruments, which may lack the responsiveness to detect meaningful differences in HRQoL. However, some studies have found that OLS regression tends to overestimate the true value of EQ-5D utilities for patients in poor health, while underestimating the true EQ-5D utilities at the upper end of the scale [16, 21–23]. Such considerations reinforce the use of a preference-based measure when assessing HRQoL in clinical trials. Nevertheless, our analysis provides an algorithm that can effectively translate FACT-P scores to generic utility values.

This study has developed an algorithm for mapping EQ-5D index scores from FACT-P. The algorithm was found to have good predictive ability, with a high degree of correlation between observed and predictive EQ-5D-based utility scores in defined subgroups of patients with mCRPC. The algorithm provides an instrument for the calculation of appropriate preference-based HRQoL scores for use in analyses of interventions for mCRPC when a generic measure is not available.
